# The promotion‐like effect of the M1‐STN hyperdirect pathway induced by ccPAS enhanced balance performances: From the perspective of brain connectivity

**DOI:** 10.1111/cns.14710

**Published:** 2024-04-14

**Authors:** Yu‐Lin Li, Jia‐Jia Wu, Xu‐Yun Hua, Mou‐Xiong Zheng, Jian‐Guang Xu

**Affiliations:** ^1^ Engineering Research Center of Traditional Chinese Medicine Intelligent Rehabilitation Ministry of Education Shanghai China; ^2^ Department of Rehabilitation Medicine, Huashan Hospital Fudan University Shanghai China; ^3^ Department of Rehabilitation Medicine, Yueyang Hospital of Integrated Traditional Chinese and Western Medicine Shanghai University of Traditional Chinese Medicine Shanghai China; ^4^ Department of Traumatology and Orthopedics, Yueyang Hospital of Integrated Traditional Chinese and Western Medicine Shanghai University of Traditional Chinese Medicine Shanghai China; ^5^ School of Rehabilitation Science Shanghai University of Traditional Chinese Medicine Shanghai China

**Keywords:** balance function, cortico‐cortical paired‐associative stimulation, hyperdirect pathway, noninvasive

## Abstract

**Aims:**

The present study aimed to explore the effect of cortico‐cortical paired‐associative stimulation (ccPAS) in modulating hyperdirect pathway and its influence on balance performance.

**Methods:**

Forty healthy participants were randomly allocated to the active ccPAS group (*n* = 20) or the sham ccPAS group (*n* = 20). The primary motor cortex and subthalamic nucleus were stimulated sequentially with ccPAS. Unlike the active ccPAS group, one wing of coil was tilted to form a 90° angle with scalp of stimulation locations for the sham ccPAS group. Magnetic resonance imaging, functional reach test (FRT), timed up and go (TUG) test, and limit of stability (LOS) test were performed, and correlation between them was also analyzed.

**Results:**

Three participants in the sham ccPAS group were excluded because of poor quality of NIfTI images. The active group had strengthened hyperdirect pathway, increased functional connectivity (FC) between orbital part of frontal cortex and bilateral precuneus, and decreased FC among basal ganglia (all *p* < 0.05). Regional network properties of triangular and orbital parts of IFG, middle cingulate cortex, and hippocampus increased. The active group performed better in FRT and LOS (all *p* < 0.05). FRT positively correlated with FC of the hyperdirect pathway (*r* = 0.439, *p* = 0.007) and FCs between orbital part of frontal cortex and bilateral precuneus (all *p* < 0.05).

**Conclusion:**

The ccPAS enhanced balance performance by promotion‐like plasticity mechanisms through the hyperdirect pathway.

## Graphical abstract

The targeted stimulation of the hyperdirect pathway (M1‐STN connectivity) with ccPAS enhanced the functional connectivity between the M1 and STN and subsequently enhanced the balance performance, highlighting the capability and efficacy of ccPAS in regulating brain connectivity associated with functional impairments.

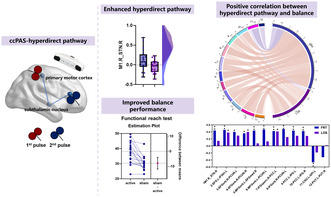

## 1 INTRODUCTION

The human brain is considered a complex but cost‐effective network that performs and completes daily functional activities in the form of brain connectivity.^1^, ^2^, ^3^ Based on the theory of spike‐timing‐dependent plasticity (STDP), brain connectivity can be strengthened/weakened and established/eliminated by repetitively and sequentially stimulating the presynaptic and postsynaptic neurons with the neuromodulation techniques.^4^, ^5^

The development of noninvasive brain stimulation not only enables us to modulate activities within brain regions, but also enables us to regulate brain connectivity via the STDP mechanism. The cortico‐cortical paired‐associative stimulation (ccPAS) consists of the repetitive application of pairs of transcranial magnetic stimulation (TMS) pulses with a defined inter‐stimulus interval (ISI) over coupled brain regions to induce STDP between them. Plenty of previous studies have proved that ccPAS can selectively strengthen and weaken brain connectivity via long‐term potentiation (LTP) and long‐term depression (LTD), respectively. The brain connectivity between the visual motor area (V5) and the primary visual area (V1) can be strengthened with ccPAS to improve the perception of coherent visual motion.^6^, ^7^ Also, the effect of ccPAS on brain connectivity depended on the stimulation order. For example, repetitive stimulation of ventral premotor cortex (PMv) before primary motor cortex (M1) increased the PMv‐M1 connectivity, while stimulation of M1 before PMv decreased it.^8^, ^9^

The deep location of the subcortical nuclei and the limited methods make it difficult to explore the feasibility of ccPAS in regulating cortical–subcortical connectivity. The deep brain stimulation (DBS) is an invasive method in stimulating deep brain nuclei, which is a common and useful intervention for treating patients with Parkinson's disease. Udupa et al. combined repetitive TMS in the M1 with DBS in the subthalamic nucleus (STN), with an ISI of 3 ms, and found that LTP‐like effects were induced for brain connectivity between M1 and STN.^10^ Currently, however, invasive STN‐DBS may be less commonly used in other brain diseases, such as stroke. Therefore, it is of great significance to propose a noninvasive method for regulating cortical–subcortical connectivity in stroke patients.

Importantly, Wang et al. proposed a novel method to find the cortical mapping target that can be used as a cortical stimulation target for TMS to indirectly and noninvasively modulate the corresponding subcortical nuclei.^11^ Specifically, the left lateral parietal cortex was defined as a cortical mapping target of the left hippocampus as it had high functional connectivity (FC) with the left hippocampus. The feasibility and effectiveness of this method have also been verified by several subsequent neuroimaging studies.^12^, ^13^, ^14^, ^15^ Therefore, the proposed method makes it possible to apply ccPAS to noninvasively modulate the cortical–subcortical connectivity and investigate its effect on functional performances related to the given brain connectivity. Our preliminary study has found that the right triangular part of the inferior frontal gyrus (rIFGtri) was a cortical mapping target of the right STN, as evidenced by that rIFGtri had high functional connectivity with the right STN (*n* = 63 healthy participants, *T* = 12.547, *p* < 0.001, as shown in Figure S1).

Balance is a basic but critical function of our daily life. It had been proved that STN‐DBS improved balance function of Parkinson's disease patients.^16^, ^17^, ^18^ The mechanism may be that the activated STN retrogradely modulated the functional activities of motor‐related cortices (such as M1) via the hyperdirect pathway.^19^, ^20^ The hyperdirect pathway is responsible for inhibition action control, and the inhibitory network is strongly right‐lateralized in the brain.^21^ Several studies had demonstrated the important role of the right M1‐STN hyperdirect pathway in inhibition controlling, which is the reason why the present study chose the right M1‐STN hyperdirect pathway.^18^, ^22^, ^23^ Based on the classic center–surround model, the cooperation among the hyperdirect, direct, and indirect pathways guarantees the accuracy and efficiency of motor programs, including balance performance.^24^

Therefore, the present study explored the feasibility of ccPAS in targeted regulating the hyperdirect pathway, as well as its effect on the balance function from the perspective of brain connectivity.

## 2 METHODS

### 2.1 Study design and participants

Based on a previous study, a total sample size of 40 healthy participants was identified.^25^ All participants were enrolled from August 2022 to August 2023 and randomly allocated to two groups: (1) the active ccPAS group and (2) the sham ccPAS group. The inclusive criteria included (1) 40–70 years old, right‐handedness; (2) no gender limitation; (3) no brain damage confirmed by MRI, including cerebral hemorrhage, ischemia, and edema; (4) no history or family history of psychiatric and psychological disorders; and (5) normal cognitive function. The exclusive criteria included (1) contraindication of MRI, including cardiac pacemaker, cardiac stent, or claustrophobia; (2) contraindication of TMS, including cochlear implant, seizure, or metal implants; (3) nursing and pregnant women; and (4) participation in any other clinical trial 1 month before. The present study was approved by the Ethics Committee of the Yueyang Hospital of Integrated Traditional Chinese and Western Medicine in accordance with the Declaration of Helsinki (No. 2022‐057). All healthy participants had signed a written informed consent before the study started.

### 2.2 Targeted ccPAS intervention

**FIGURE 1:**
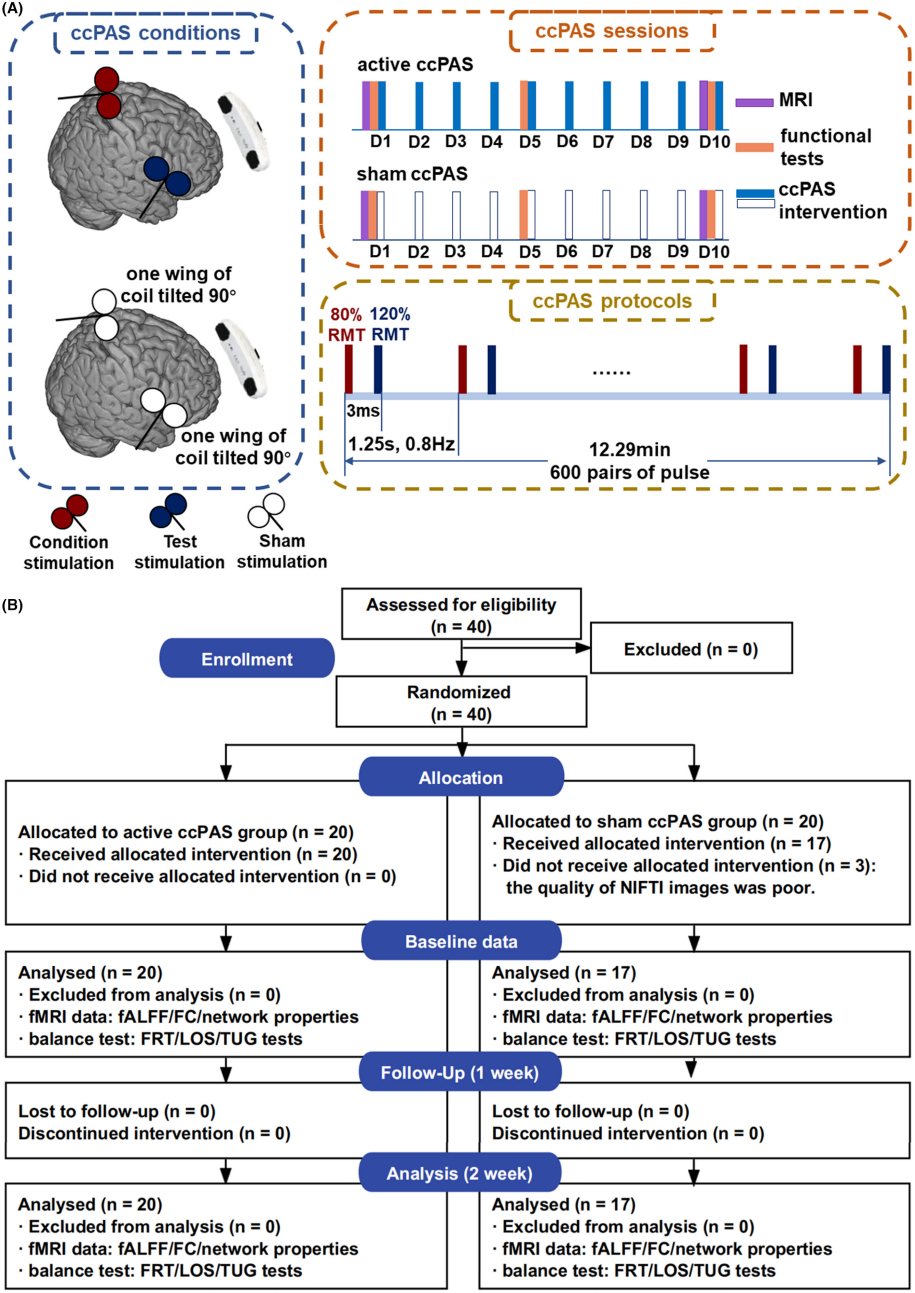
The design and CONSORT diagram. (A) Trial design. (B) CONSORT diagram: enrollment, allocation, baseline data, follow‐up, and analysis. ccPAS, cortico‐cortical paired‐associative stimulation; D, day; RMT, resting motor threshold.

The ccPAS intervention was delivered for 10 consecutive days. Before ccPAS, participants were asked to complete a “safety screening questionnaire for rTMS” first to screen the risk of adverse events during ccPAS, as shown in Table S1.^26^ The ccPAS was delivered with the MagVenture stimulator (MagPro R3.0; Denmark) connected to two coils (Cool D‐B80 and MC‐B65‐HO). The healthy participants allocated to the active ccPAS group received the ccPAS intervention with the following parameters (Figure 1A): 0.8 Hz, 600 pairs of pulses, ISI of 3 ms, and 12.29 min total.^27^ It had been proved that TMS with a frequency lower than 0.9 Hz did not affect the excitability of the cortex, thereby avoiding the confounding effects induced by high frequency on ccPAS.^10^ In addition, a sufficiently short ISI did not influence the functional connectivity between two cortical brain regions, such as the right M1 and rIFGtri.^28^, ^29^ The stimulation intensity of coils 1 and 2 was 80% resting motor threshold (RMT) and 120% RMT, respectively.^26^, ^30^ Coil 1 was placed on the subject‐specific leg‐M1. Specifically, coil 1 was first placed over the vertex (Cz) and then moved to the cortical location in small increments to generate a maximum motor‐evoked potential (MEP) response for the tibialis anterior (TA) at the intensity of RMT, known as a hotspot of TA. Coil 2 was placed on the subject‐specific rIFGtri. To be specific, the voxel within the rIFGtri with the maximum functional connectivity with the right STN was determined first, and then, a 15‐mm‐radius sphere with this voxel as its center was drawn in the individual brain space, referred to as the cortical mapping target of the right STN.^11^ The consistency of locations of stimulation targets was supervised by the Brainsight TMS Navigation system (UK) (Figure 1A).

The healthy participants assigned to the sham ccPAS group received the ccPAS intervention with the above parameters, except for the direction of coils. To be specific, one wing of the coil was tilted to form an angle of 90° with the scalp of stimulation locations to reduce magnetic stimulation to the cortices to the most extent.^31^

### 2.3 MR data acquisitions and preprocessing

The MR images were collected with the MAGNETOM Verio 3.0 T (Siemens Healthcare, Erlangen, Germany) before and after ten consecutive days of targeted ccPAS interventions. Resting‐state fMRI images were acquired by using the gradient‐recalled echo–echo‐planar imaging (GRE‐EPI) sequence with the following parameters: repetition time (TR) = 3000 ms, echo time (TE) = 30 ms, flip angle (FA) = 90°, field of view (FOV) = 256 × 256 mm, timepoints = 200, slice thickness = 3 mm, slice number = 43, and matrix size = 64 × 64. The T1‐weighted structural images were obtained by using magnetization‐prepared rapid gradient echo (MPRAGE) sequence with the following parameters: TR = 1900 ms, TE = 2.93 ms, inversion time = 900 ms, slice thickness = 1 mm, FA = 9°, FOV = 256 × 256 mm, and matrix size = 256 × 256.

The DICOM images were converted to NIfTI images with the dcm2nii software (http://www.mricro.com). The original NIfTI images were visually checked for defects and artifacts by two independent researchers. The anterior commissure of original NIfTI images was manually reoriented to the origin of the 3D Montreal Neurological Institute (MNI) space. The fMRI data were preprocessed with the following steps with the RESTplus toolbox, including removal of the first 10 volumes, slice timing, realignment, normalization with DARTEL, smoothing with a 6‐mm Gaussian kernel, detrending, regression covariates (including global mean signal, CSF signal, white matter signal, and head motion), and filter (0.01–0.08 Hz).^32^, ^33^ The normalized NIfTI images were visually checked again to exclude the poor‐normalization images from further data analysis. Participants would be excluded if their head movement was greater than 2.5 mm displacement or 2.5° rotation.^34^

### 2.4 The calculation of fractional amplitude of low‐frequency fluctuation (fALFF)

The ALFF is defined as the sum of the amplitude of the power spectrum of each voxel over the low‐frequency range (0.01–0.08 Hz), reflecting the strength of local spontaneous activity of the neuron at the resting state.^35^ The fractional ALFF (fALFF) is more specific, sensitive, and reliable for measuring regional spontaneous activity than ALFF by measuring the ratio of the ALFF within a low‐frequency range (0.01–0.08 Hz) to the whole frequency range.^36^ Therefore, bandpass filter was not performed for the calculation of fALFF. To improve the data normality, for each voxel, the mean fALFF value of all the voxels in the whole brain was subtracted from the fALFF voxel and then divided by the standard deviation of fALFF values of all voxels; then, the zfALFF was subsequently obtained.

### 2.5 The calculation of functional connectivity, construction of individual functional brain networks, and graph theoretical analysis

In addition to the automated anatomical labeling (AAL) atlas, bilateral STNs (https://fsl.fmrib.ox.ac.uk/fsl/fslwiki/Atlases) were added to constitute a new brain atlas, including 92 regions of interest (ROIs). The correlation of mean time‐series blood oxygenation level‐dependent (BOLD) signals among 92 ROIs was calculated by the Pearson correlation analysis and then normalized to improve its normality, constituting an individual functional connectivity matrix *R*_*ij*_ (*i* = 1,2,3…*N*, *j* = 1,2,3…*N*, *N* = 92). The basal ganglia were further subdivided into caudate, putamen, thalamus, STN, substantia nigra pars reticulata (SN_pr), substantia nigra pars compacta (SN_pc), internal globus pallidus (GPi), and external globus pallidus (GPe) in bilateral hemispheres. The correlation among these 16 ROIs was also calculated and normalized. The above ROI‐wise functional connectivity was calculated with the REST toolbox (v1.9).^37^

The network density was set at 0.05 to 0.5 with the step of 0.01 (0.05:0.01:0.5),^38^, ^39^ and the matrix *R*_*ij*_ of each participant was then thresholded with the network density, resulting in an unweighted, nondirectional binarized matrix referred to as a graph. Finally, the global and regional network properties of the graph were quantified with the graph theoretical network analysis (GRETNA) toolbox (v2.0).^40^ The global network properties, including assortativity, hierarchy, global efficiency (*E*_*glob*_), local efficiency (*E*_*loc*_), clustering coefficient (*C*_*p*_), characteristic path length (*L*_*p*_), small‐worldness index, and synchronization, and regional network properties, including nodal *C*_*p*_, nodal *Lp* (*NL*_*p*_), nodal efficiency, nodal *E*_*loc*_, betweenness centrality (BC), and degree, were analyzed in the present study. Overall, the global and regional network properties indicated efficiency of information communication at global and local levels, respectively; the more detailed definition, calculation, and physiological significance were presented in our previous studies.^41^, ^42^, ^43^

### 2.6 The evaluation of balance tests

Three balance tests were evaluated in the present study, including the functional reach test (FRT), limit of stability (LOS) test, and timed up and go (TUG) test. The FRT is a reliable test for evaluating the dynamic balance function for the elderly, and patients with stroke or Parkinson's disease.^44^, ^45^, ^46^, ^47^ The healthy participant was asked to reach forward as far as possible during the test without losing their balance; the maximal distance (cm) was recorded.^48^ The greater the distance, the better the balance function.

Similar to the FRT, LOS test also evaluates the dynamic balance function. The healthy participant was instructed to intentionally move their center of gravity (COG) to eight different directions (forward, forward–right, right, backward–right, backward, backward–left, left, and forward–left) on a stable platform (ProKin‐252; TecnoBody, Bergamo, Italy) and maintain that position for 8 s.^49^ The maximal distance was expressed as the maximum excursion (MXE). The greater MXE also indicates a better dynamic balance function.

TUG test measures dynamic balance and functional mobility for the elderly and patients with neurological diseases. The total time taken to complete the whole process, including standing up, walking a distance of 3 m, turning, walking back, and sitting down again, would be recorded as the score of the TUG test.^50^, ^51^ TUG score is correlated with the risk of falls, and the greater the score, the higher the risk.

### 2.7 Statistical analysis

The statistical analysis was performed with the SPSS Statistics V25 (IBM Corporation, Chicago, IL). Normally and non‐normally distributed data were described as mean ± standard deviation (SD) and median (min, max). The baseline data and differences in outcomes before and after ccPAS intervention between the active ccPAS and sham ccPAS groups were analyzed with the independent samples *T*‐test. The multiple comparison was corrected with the false discovery rate (FDR) method with *p* < 0.05. The enumeration data were expressed as *n* (%) and analyzed with the chi‐square test. The correlation between FC/network properties and balance tests was analyzed using Pearson's or Spearman's correlation analyses. The significant level *α* was set at 0.05 (two‐tailed). The results were visualized with the GraphPad Prism 9.0 (San Diego, USA, https://www.graphpad.com/), MRIcroGL (version 11), RStudio (V 4.2.0), and BrainNet Viewer (http://www.nitrc.org/projects/bnv/).

## 3 RESULTS

### 3.1 Demographic and baseline characteristics

A total of 40 healthy participants were enrolled. Three participants in the sham ccPAS group were excluded because of poor quality of NIfTI images, as shown in Figure 1B. The mean (SD) age of the active and sham ccPAS groups was 55.75 (5.04) and 54.71 (6.06), respectively. There was a similar distribution of gender (*χ* ^*2*^ = 0.288, *p* = 0.591). The inter‐group difference of weight (*T* = −1.868, *p* = 0.070), height (*T* = −1.160, *p* = 0.254), BMI (*T* = −1.804, *p* = 0.080), FRT (*T* = −0.117, *p* = 0.908), TUG test (*T* = 0.721, *p* = 0.476), and LOS test (*T* = 1.247, *p* = 0.221) was not found, as shown in Table 1.

**TABLE 1:** Demographic characteristics and pre‐treatment baseline data.

	Active ccPAS group	Sham ccPAS group	Mean difference (95% CI)	*T*/*χ* ^*2*^	*p*
*n*	20	17	—	—	—
Age (years)	55.75 ± 5.04	54.71 ± 6.06	1.044 (−2.658, 4.746)	0.573	0.571
Gender					
Female	10 (50%)	10 (59%)	—	0.288	0.591
Male	10 (50%)	7 (41%)	—		
Weight, kg	64.05 ± 11.54	69.82 ± 5.83	−5.774 (−12.049, 0.502)	−1.868	0.070
Height, m	1.63 ± 0.06	1.65 ± 0.06	−0.023 (−0.064, 0.018)	−1.160	0.254
BMI, kg/m^2^	24.10 ± 3.17	25.67 ± 1.79	−1.568 (−3.332, 0.196)	−1.804	0.080
FRT	28.65 ± 7.15	28.89 ± 4.76	−0.238 (−4.372, 3.896)	−0.117	0.908
TUG test	8.56 ± 0.81	8.36 ± 0.88	0.199 (−0.362, 0.761)	0.721	0.476
LOS test	67.43 ± 8.32	63.89 ± 8.83	3.537 (−2.233, 9.306)	1.247	0.221

*Note*: Data were described as mean ± standard deviation or *n* (%).

Abbreviations: BMI, body mass index; ccPAS, cortico‐cortical paired‐associative stimulation; CI, confidence interval; FRT, functional reach test; LOS, limit of stability; TUG, timed up and go.

### 3.2 The results of fALFF analysis

**FIGURE 2:**
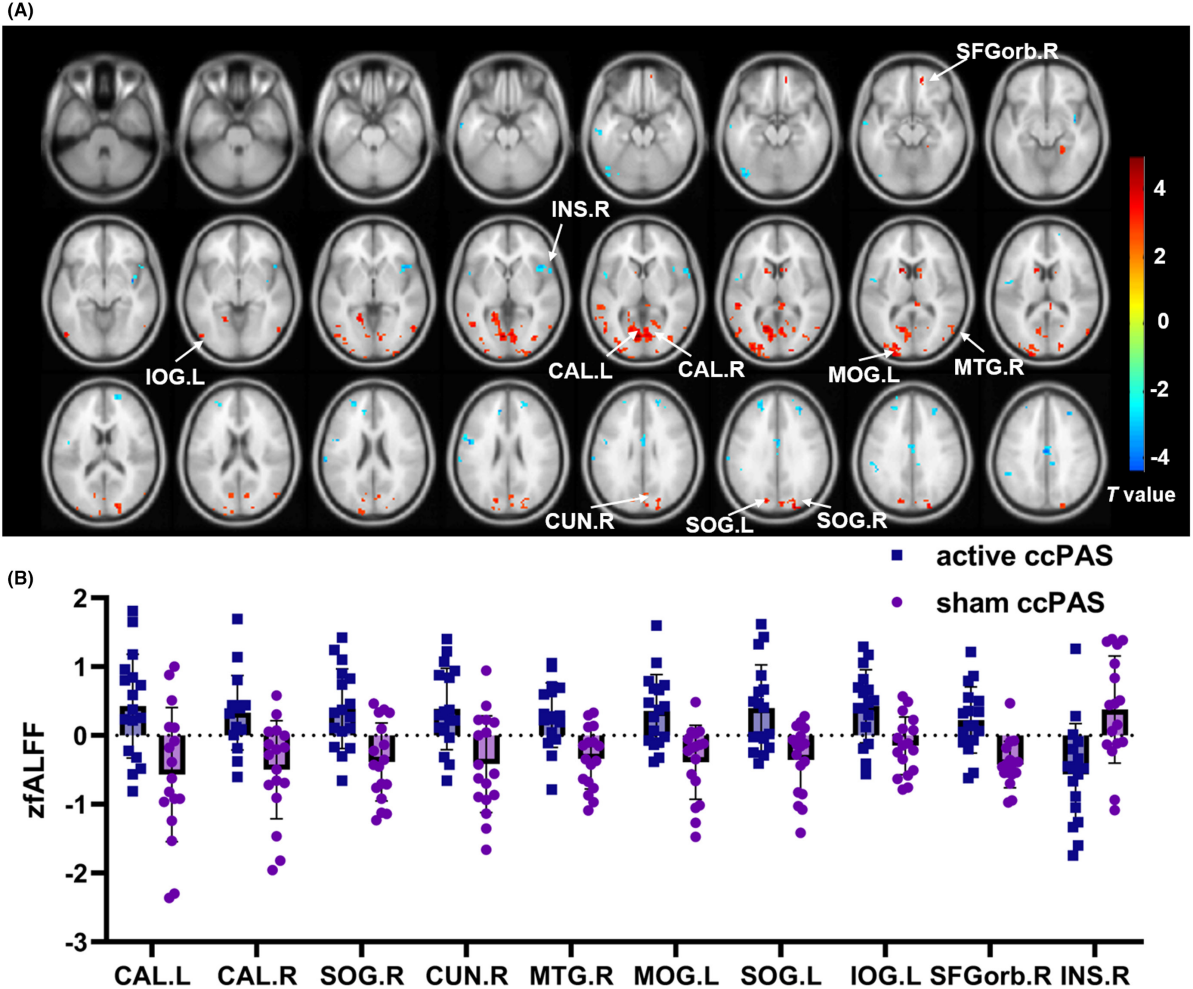
The inter‐group comparison of zfALFF. Compared with the sham ccPAS group, the active ccPAS group had higher zfALFF in bilateral calcarine, bilateral SOG, right cuneus, right MTG, left MOG, left IOG, right SFGorb, and lower zfALFF in right insula, as shown in (A) and (B). ccPAS, cortico‐cortical paired‐associative stimulation; CAL, calcarine; SOG, superior occipital gyrus; CUN, cuneus; MTG, middle temporal gyrus; MOG, middle occipital gyrus; IOG, inferior occipital gyrus; SFGorb, orbital part of superior frontal gyrus; INS, insula; L, left; R, right.

Compared with the sham ccPAS group, the active ccPAS group had higher zfALFF in bilateral calcarine, bilateral superior occipital gyri (SOG), right cuneus, right middle temporal gyrus (MTG), left middle occipital gyrus (MOG), left inferior occipital gyrus (IOG), right orbital part of superior frontal gyrus (SFGorb), and lower zfALFF in the right insula, as shown in Table S2 and Figure 2.

### 3.3 The results of ROI‐wise functional connectivity analysis

**FIGURE 3:**
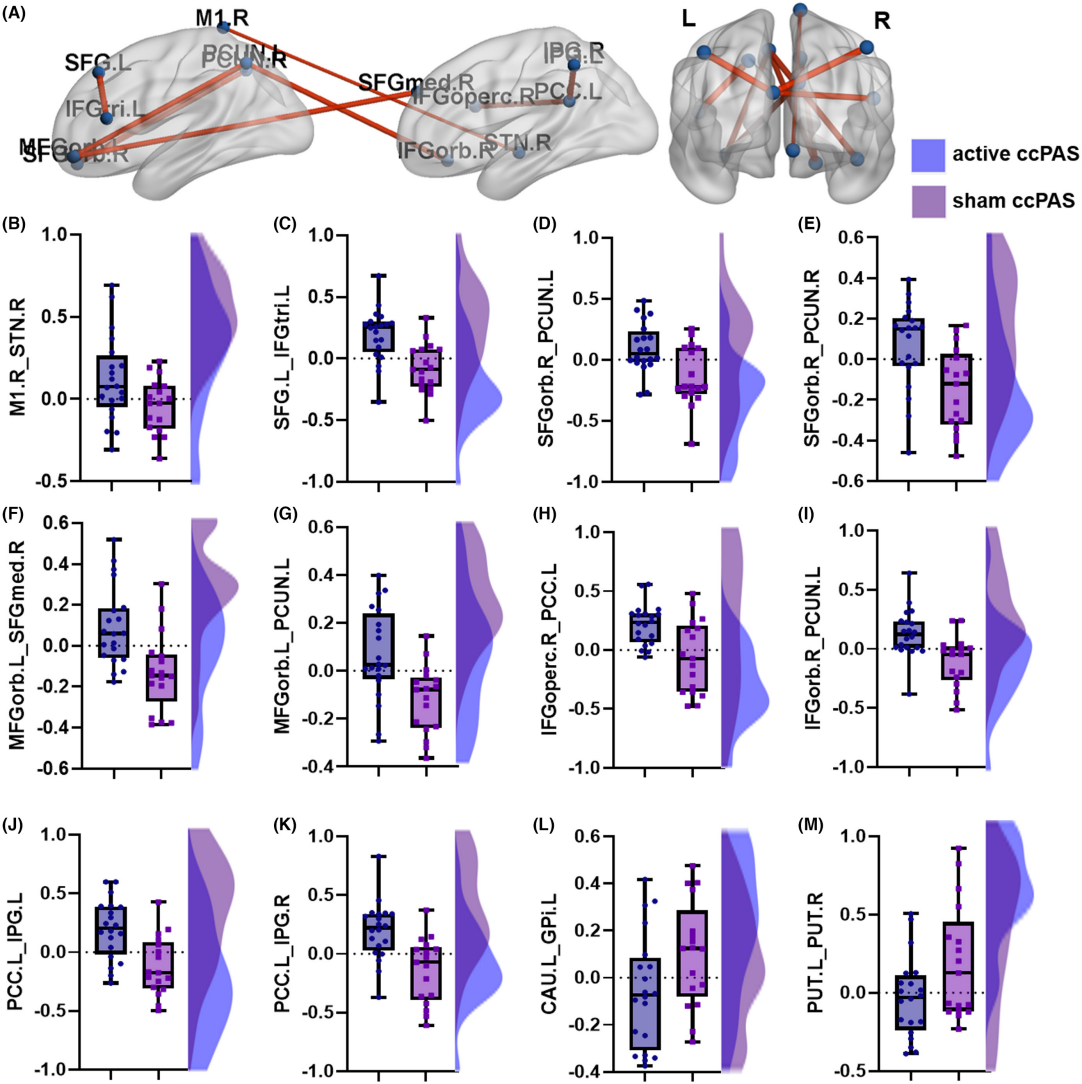
The inter‐group comparison of the ROI‐wise functional connectivity. The inter‐group differences of ROI‐wise functional connectivity (FC) are shown in (A); red and blue lines indicated increased FC and decreased FC, respectively. The active ccPAS group had higher (B–K, all *p* < 0.05) and lower (L, M, all *p* < 0.05) ROI‐wise FCs than the sham ccPAS group after 10 consecutive days of ccPAS intervention. CAU, caudate; ccPAS, cortico‐cortical paired‐associative stimulation; GPi, internal globus pallidus; IFGoperc, opercular part of inferior frontal gyrus; IFGorb, orbital part of inferior frontal gyrus; IFGtri, triangular part of inferior frontal gyrus; IPG, inferior parietal gyrus; L, left; MFGorb, orbital part of middle frontal gyrus; PCC, posterior cingulate cortex; PCUN, precuneus; PUT, putamen; R, right; SFG, superior frontal gyrus; SFGmed, medial part of superior frontal gyrus; SFGorb, orbital part of superior frontal gyrus; STN, subthalamic nucleus.

Compared with the sham ccPAS group, the active ccPAS group had increased ROI‐wise FC between right M1 and right STN (hyperdirect pathway, *T* = 2.164, *p* = 0.037), left SFG and left IFGtri (*T* = 3.898, *p* = <0.001), right SFGorb and bilateral precunei (left: *T* = 3.151, *p* = 0.003; right: *T* = 3.180, *p* = 0.003), left orbital part of middle frontal gyrus (MFGorb) and right medial part of SFG (SFGmed) (*T* = 3.435, *p* = 0.002), left MFGorb and left precuneus (*T* = 3.318, *p* = 0.002), right opercular part of inferior frontal gyrus (IFGoperc) and left PCC (*T* = 3.387, *p* = 0.002), right orbital part of inferior frontal gyrus (IFGorb) and left precuneus (*T* = 3.418, *p* = 0.002), and left PCC and bilateral inferior parietal gyri (IPG, left: *T* = 3.803, *p* = 0.001; right: *T* = 3.857, *p* < 0.001), and decreased FC between left caudate and left internal globus pallidus (GPi) (*T* = −2.085, *p* = 0.044) and bilateral putamina (*T* = −2.181, *p* = 0.036) after ten consecutive days of ccPAS intervention, as shown in Table S3 and Figure 3.

### 3.4 The results of global network analysis

There was no inter‐group difference of global network properties including assortativity (*T* = −1.060, *p* = 0.296), hierarchy (*T* = 2.034, *p* = 0.050), global efficiency (*T* = −0.532, *p* = 0.598), local efficiency (*T* = 0.772, *p* = 0.445), *Cp* (*T* = 0.531, *p* = 0.599), *Lp* (*T* = 0.454, *p* = 0.653), *sigma* (*T* = 1.471, *p* = 0.150), and synchronization (*T* = −0.642, *p* = 0.525), as shown in Table S4 and Figure S2.

### 3.5 The results of regional network analysis

**FIGURE 4:**
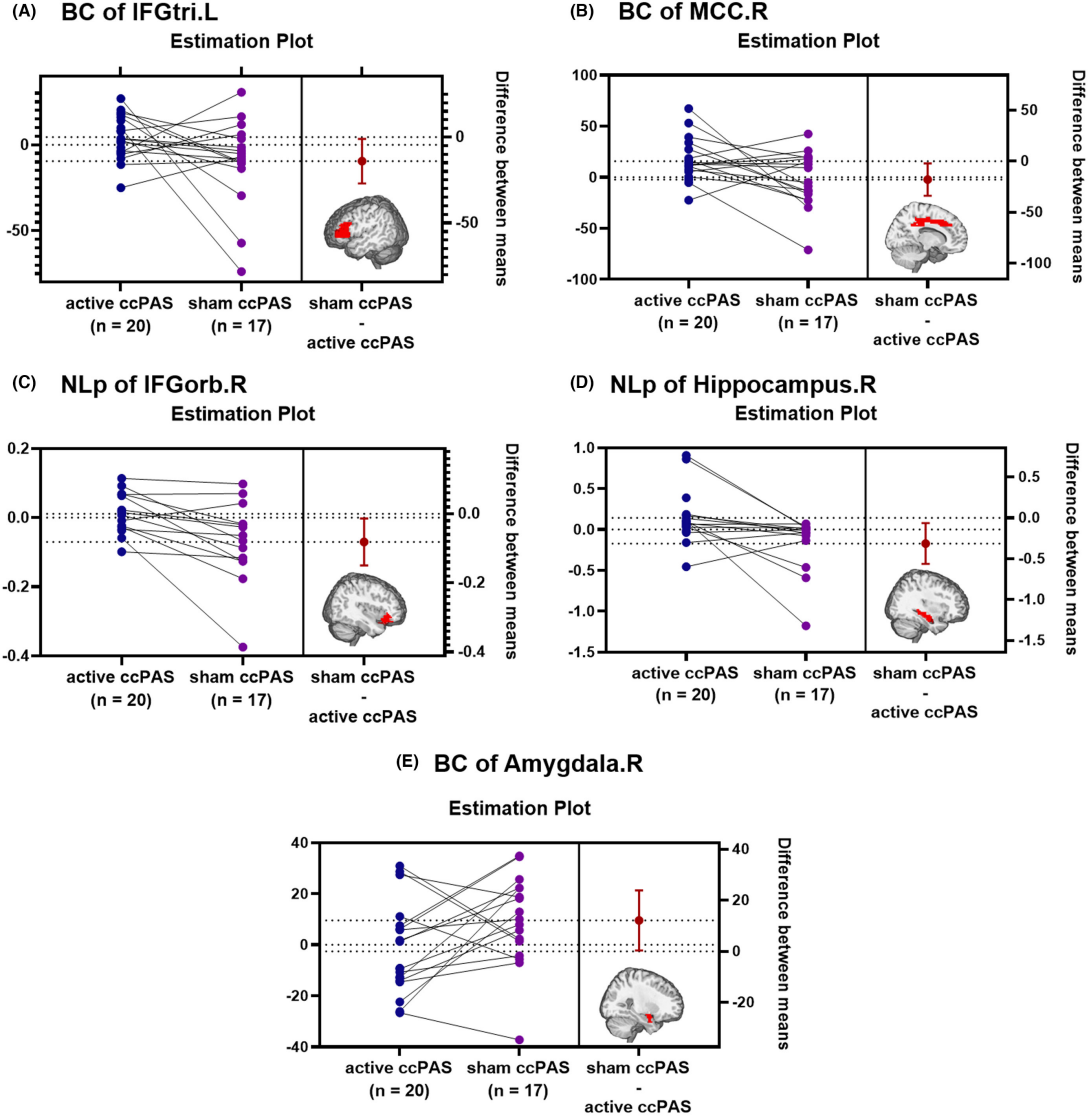
The inter‐group comparison of the regional network properties. The active ccPAS group had higher BC in left IFGtri (A) and right MCC (B), higher *NL*_*p*_ in right IFGorb (C) and right hippocampus (D), and lower BC in right amygdala (E) than the sham ccPAS group (all *p* < 0.05). BC, betweenness centrality; IFGorb, orbital part of inferior frontal gyrus; IFGtri, triangular part of inferior frontal gyrus; L, left; MCC, middle cingulate cortex; NL_p_, nodal characteristic path length; R, right.

The active ccPAS group showed higher BC in left IFGtri (*T* = 2.206, *p* = 0.034) and right MCC (*T* = 2.321, *p* = 0.026), higher *NL*_*p*_ in right IFGorb (*T* = 2.519, *p* = 0.018) and right hippocampus (*T* = 2.582, *p* = 0.015), and lower BC in right amygdala (*T* = −2.096, *p* = 0.043) than the sham ccPAS group, as shown in Table S5 and Figure 4.

### 3.6 The results of balance tests

The results are presented in Table 2 and Figure S3. The active ccPAS group had better performance in FRT and LOS tests than the sham ccPAS group with mean differences (95% CIs) of 7.850 (3.964, 11.735) and 12.243 (7.802, 16.684), respectively. There was no inter‐group difference of TUG test (*T* = −0.950, *p* = 0.349).

**TABLE 2:** The inter‐group comparison of the balance tests after ccPAS intervention.

Clinical outcomes	Active ccPAS group	Sham ccPAS group	Mean difference (95% CI)	*T*	*p*
FRT	38.185 ± 6.717	30.335 ± 4.479	7.850 (3.964, 11.735)	4.101	<0.001
LOS test	79.709 ± 5.315	67.466 ± 7.916	12.243 (7.802, 16.684)	5.596	<0.001
TUG test	8.151 ± 0.797	8.402 ± 0.808	−0.251 (−0.789, 0.286)	−0.950	0.349

Abbreviations: ccPAS, cortico‐cortical paired‐associative stimulation; CI, confidence interval; FRT, functional reach test; LOS, limit of stability; TUG, timed up and go.

### 3.7 The results of Pearson's correlation analysis

#### 3.7.1 The correlation between zfALFF and balance tests

There were positive correlations between FRT and zfALFF of brain regions including right SFGorb, bilateral calcarine, right cuneus, bilateral SOG, left MOG, left IOG, and right MTG (all *p* < 0.05). The LOS also had positive relationships with zfALFF of brain regions including bilateral calcarine, right cuneus, bilateral SOG, left MOG, and right MTG (all *p* < 0.05), as shown in Table S6 and Figure S4. On the contrary, the FRT was negatively correlated with zfALFF of right insula (*r* = −0.490, *p* = 0.002). There was no correlation between TUG and zfALFF (*p* > 0.05).

#### 3.7.2 The correlation between ROI‐wise FC and balance tests

**FIGURE 5:**
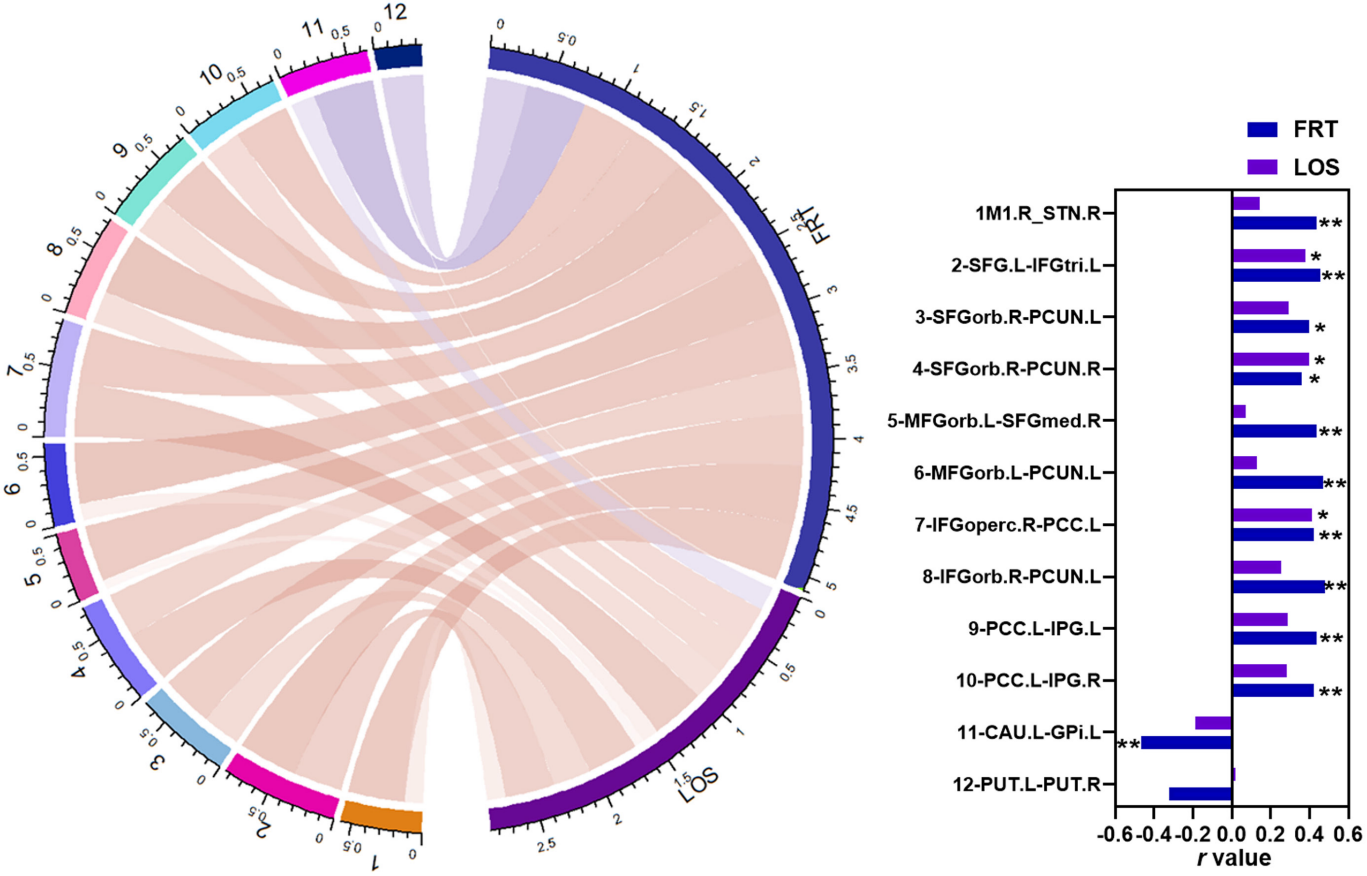
The correlations between ROI‐wise FC and balance tests. Red and blue lines in the left circle indicated positive and negative correlations, respectively. The thicker the line, the darker the color and the greater the *r* values. CAU, caudate; FRT, functional reach test; GPi, internal globus pallidus; IFGoperc, opercular part of inferior frontal gyrus; IFGorb, orbital part of inferior frontal gyrus; IFGtri, triangular part of inferior frontal gyrus; IPG, inferior parietal gyrus; L, left; LOS, limit of stability; MFGorb, orbital part of middle frontal gyrus; PCC, posterior cingulate cortex; PCUN, precuneus; PUT, putamen; R, right; SFG, superior frontal gyrus; SFGmed, medial part of superior frontal gyrus; SFGorb, orbital part of superior frontal gyrus; STN, subthalamic nucleus. *p < 0.05; **p < 0.01.

The FRT was positively correlated with the ROI‐wise FC between the right M1 and right STN (hyperdirect pathway), left SFG and left IFGtri, right SFGorb and bilateral precunei, left MFGorb and right SFGmed, left MFGorb and left precuneus, right IFGoperc and left PCC, right IFGorb and left precuneus, and left PCC and bilateral IPG (all *p* < 0.05). The LOS had positive relationships with FC between left SFG and left IFGtri, right SFGorb and right precuneus, and right IFGoperc and left PCC (all *p* < 0.05), and the FRT had a negative correlation with the FC between left caudate and left GPi (*r* = −0.464, *p* = 0.004). The above results are shown in Table S7 and Figure 5.

## 4 DISCUSSION

The present study found that cortical–subcortical connectivity (hyperdirect pathway, M1‐STN) can be specifically modulated by noninvasive ccPAS, and modulating the hyperdirect pathway with ccPAS resulted in a concomitant enhancement in balance performance. In addition, ccPAS of the hyperdirect pathway activated visual‐related cortices, including bilateral calcarine, bilateral SOG, right cuneus, and left MOG and IOG, and increased the functional connectivity between visual‐ and executive control‐related cortices. Also, the above increased functional activities and connectivities had positive relationships with enhanced balance performances. The ccPAS of the hyperdirect pathway did not change the information communication efficiency at the global level, but increased the regional communication efficiency of brain regions, including right MCC and IFGorb, and hippocampus. The present study provided evidence that cortical–subcortical connectivity can be targeted to noninvasively modulate by ccPAS. Consistent with the characteristics of the brain working in the form of connectivity, targeted ccPAS of the hyperdirect pathway may be a capable intervention method in treating patients with balance dysfunction, such as stroke.

### 4.1 The LTP‐like effect of the M1‐STN hyperdirect pathway induced by ccPAS enhanced balance performances

A previous study found that the M1‐STN hyperdirect pathway could be regulated by the sequential stimulation of M1 and STN with noninvasive TMS and invasive DBS, respectively.^10^ On the contrary, the present study aimed to explore the feasibility of ccPAS in noninvasively modulating the same pathway. The combination of ccPAS and fMRI provides the advantage of investigating brain connectivity of the motor cortex and subcortical nuclei. Interestingly, we found that the LTP‐like effect was demonstrated in the present study, as evidenced by that the M1‐STN hyperdirect pathway was strengthened after ccPAS intervention.

As the hyperdirect pathway bypasses the striatum, it can rapidly convey the signals of motor planning to the output structures (SN_pr and GPi) of basal ganglia to extensively inhibit activities of cortices related to the competitive motor program.^52^ Therefore, the accuracy and efficiency of the targeted motor programs can be ensured, such as functional performances related to balance. The findings of the present study also demonstrated that the LTP‐like effect induced by targeted ccPAS of the hyperdirect pathway enhanced the balance performance of healthy subjects. Previous studies found that the LTP‐like effect of the M1‐STN hyperdirect pathway could be promoted by increasing activation of N‐methyl‐D‐aspartate (NMDA) receptors, one subset of the excitable glutamic acid receptor.^53^ Also, as structural and functional changes are mutually causal, exploring the effect of target ccPAS of the M1‐STN hyperdirect pathway on its structural connectivity and the correlation between structural and functional connectivity is necessary to be explored in the future.^54^

### 4.2 Targeted neuromodulation of the M1‐STN pathway also increased functional activities and connectivity of brain regions related to balance function

In addition to enhancement of the M1‐STN hyperdirect pathway, the functional activities and connectivity of brain regions related to balance function were also increased after targeted neuromodulation.

Consistent with previous studies, the present study also found that ccPAS of the hyperdirect pathway increased the functional activities of brain regions mainly located in the visual‐ and executive control‐related cortices, as well as functional connectivity between them.^55^, ^56^ Based on the subnetworks divided by Yeo et al., the visual subnetwork includes the brain regions including bilateral calcarine, cuneus, precuneus, occipital gyrus, posterior cingulate cortex, and posterior parietal cortex.^57^, ^58^ These brain regions are primarily responsible for receiving and integrating visual information and then transferring it to other brain structures for analysis.^59^ For example, completing the balance performances relies on the cooperation between visual‐ and executive control‐related cortices, including the frontal lobe. Plenty of studies have demonstrated the importance of the prefrontal cortex as a higher‐order cognitive brain structure for performing balance activities. Fougère et al. found that the prefrontal cortex was activated both during real and imagined locomotion.^60^ The positive relationships indicated that the satisfied cooperation between brain regions permits us to perform balance better. However, the disease‐related poor cooperation between brain regions compromises both brain network and behavioral performances.^61^ The effect of targeted neuromodulation of brain connectivity among visual‐ and executive control‐related cortices on balance function could also be explored in future studies.

## 5 CONCLUSIONS

The targeted neuromodulation of the M1‐STN hyperdirect pathway with noninvasive ccPAS enhanced its strength and caused correlated improvement in balance performances. In addition, targeted neuromodulation also increased functional activities and connectivity of brain regions related to balance function. The above findings proved the feasibility and efficiency of noninvasive ccPAS in regulating the cortical–subcortical connectivity, providing evidence for treating balance dysfunction from the perspective of brain connectivity.

## 6 LIMITATIONS

Exploring the relationship between functional and structural changes following ccPAS intervention could be a valuable future research direction. In addition, with the development of neuroimaging techniques, it is an absorbing topic to investigate the effects of state‐dependent neuromodulation on balance functions for stroke patients. Also, the use of multiple parcellation templates, including functional templates, allows us to compare and integrate the information provided by different templates, thereby leading to a more comprehensive understanding of brain functional networks related to different conditions.

## AUTHOR CONTRIBUTIONS

Conceptualization: Xu‐Yun Hua, Mou‐Xiong Zheng, and Jia‐Jia Wu. Methodology: Xu‐Yun Hua, Mou‐Xiong Zheng, and Jia‐Jia Wu. Validation: Jia‐Jia Wu. Formal analysis: Xu‐Yun Hua, Mou‐Xiong Zheng, Jia‐Jia Wu, and Yu‐Lin Li. Writing – original draft: Yu‐Lin Li and Mou‐Xiong Zheng. Writing – review and editing: Jian‐Guang Xu. All authors read and approved the final manuscript.

## FUNDING INFORMATION

This work was supported by the National Natural Science Foundation of China (Grant Nos. 81871836, 82172554, 82272583, and 82272589). National Key R&D Program of China (Grant Nos. 2018YFC2001600 and 2018YFC2001604). Shanghai Youth Top Talent Development Plan. Shanghai Rising‐Star Program (Grant No. 23QA1409200). Shanghai Talent Development Fund (Grant No. 2021074). Shanghai “Rising Stars of Medical Talent”—Distinguished Young Medical Talent Program. Shanghai Science and Technology Committee (Grant No. 22010504200). Shanghai Health Care commission (Grant No. 2022JC026).

## CONFLICT OF INTEREST STATEMENT

The authors have no relevant financial or non‐financial interests to disclose.

## INFORMED CONSENT

Informed consent was obtained from all individual participants included in the study.

## Supporting information


Appendix S1.

## Data Availability

Jian‐Guang Xu is the guarantor of this work and, as such, has full access to all the data in the study and takes responsibility for the integrity of the data and the accuracy of the data analysis. All the data supporting our findings are available on reasonable request.
