# Supplementary material for: The promotion‐like effect of the M1‐STN hyperdirect pathway induced by ccPAS enhanced balance performances: From the perspective of brain connectivity
Source: CNS Neurosci Ther. 2024 Apr 14;30(4):e14710. doi: 10.1111/cns.14710 (PMC11016345; doi:10.1111/cns.14710)
Supplement: Supplementary file 1 — Appendix S1. [file CNS-30-e14710-s001.docx]

**Supplemental Materials**

# Title page

**The promotion-like effect of the M1-STN hyperdirect pathway induced by ccPAS enhanced balance performances: from the perspective of brain connectivity**

Yu-Lin Li, M.D., Ph.D.^1,2^*, Jia-Jia Wu, M.D., Ph.D.^3^*, Xu-Yun Hua, M.D., Ph.D.^4^#, Mou-Xiong Zheng, M.D., Ph.D.^4^#, Jian-Guang Xu, M.D., Ph.D.^1,3,5^#

1. Engineering Research Center of Traditional Chinese Medicine Intelligent Rehabilitation, Ministry of Education, Shanghai, 201203, China.
2. Department of Rehabilitation Medicine, Huashan Hospital, Fudan University, Shanghai, 200040, China.
3. Department of Rehabilitation Medicine, Yueyang Hospital of Integrated Traditional Chinese and Western Medicine, Shanghai University of Traditional Chinese Medicine, Shanghai, 200437, China.
4. Department of Traumatology and Orthopedics, Yueyang Hospital of Integrated Traditional Chinese and Western Medicine, Shanghai University of Traditional Chinese Medicine, Shanghai, 200437, China.
5. School of Rehabilitation Science, Shanghai University of Traditional Chinese Medicine, Shanghai, 201203, China.

*** These authors contributed equally to this work.**

**# Corresponding authors:**

Correspondence to:

1. Jian-Guang Xu, M.D., Ph.D.

Engineering Research Center of Traditional Chinese Medicine Intelligent Rehabilitation, Ministry of Education, 201203, Shanghai, China.

No.1200 Cailun Road, Shanghai, China

Tel: +86-21-51322091

Fax: +86-21-51322042

E-mail: [xjg@shutcm.edu.cn](mailto:xjg@shutcm.edu.cn)

1. Mou-Xiong Zheng, M.D., Ph.D.

Department of Traumatology and Orthopedics, Yueyang Hospital of Integrated Traditional Chinese and Western Medicine, Shanghai University of Traditional Chinese Medicine, Shanghai, 200437, China.

No.110 Ganhe Road, Shanghai, China

Tel: +86-21-51322091

Fax: +86-21-51322042

E-mail: [zhengmouxiong@shutcm.edu.cn](mailto:zhengmouxiong@shutcm.edu.cn)

1. Xu-Yun Hua, M.D., Ph.D.

Department of Traumatology and Orthopedics, Yueyang Hospital of Integrated Traditional Chinese and Western Medicine, Shanghai University of Traditional Chinese Medicine, Shanghai, 200437, China.

No.110 Ganhe Road, Shanghai, China

Tel: +86-21-51322091

Fax: +86-21-51322042

E-mail: [huaxuyun@shutcm.edu.cn](mailto:huaxuyun@shutcm.edu.cn)


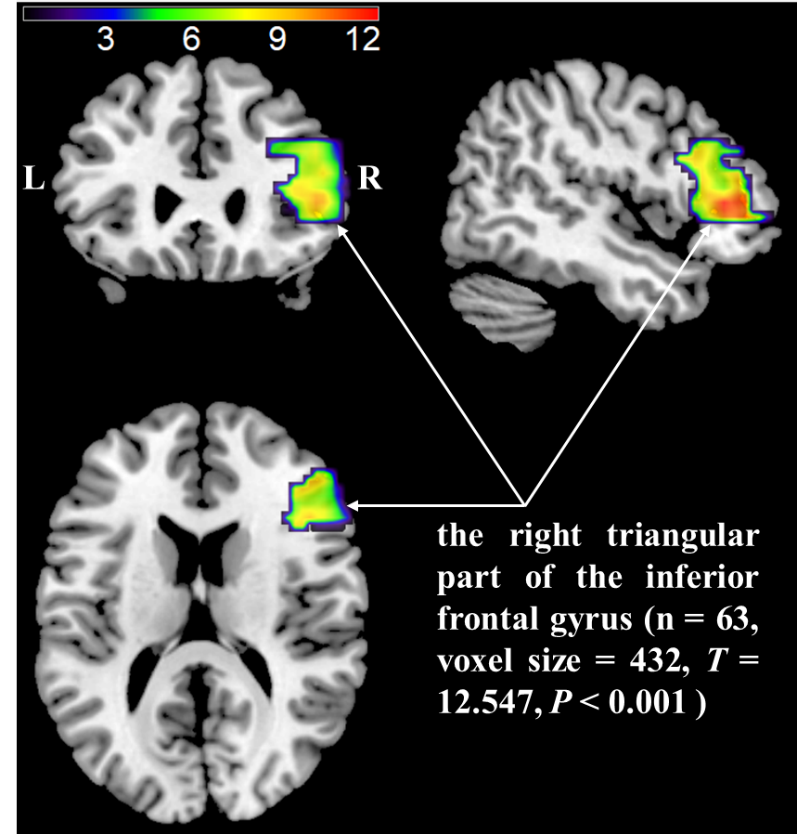


**Figure S1. The cortical mapping target of the right STN.** The right triangular part of the inferior frontal gyrus was a cortical mapping target of the right STN, as evidenced by that rIFGtri had high functional connectivity with the right STN. *STN, subthalamic nucleus. L, left. R, right*.


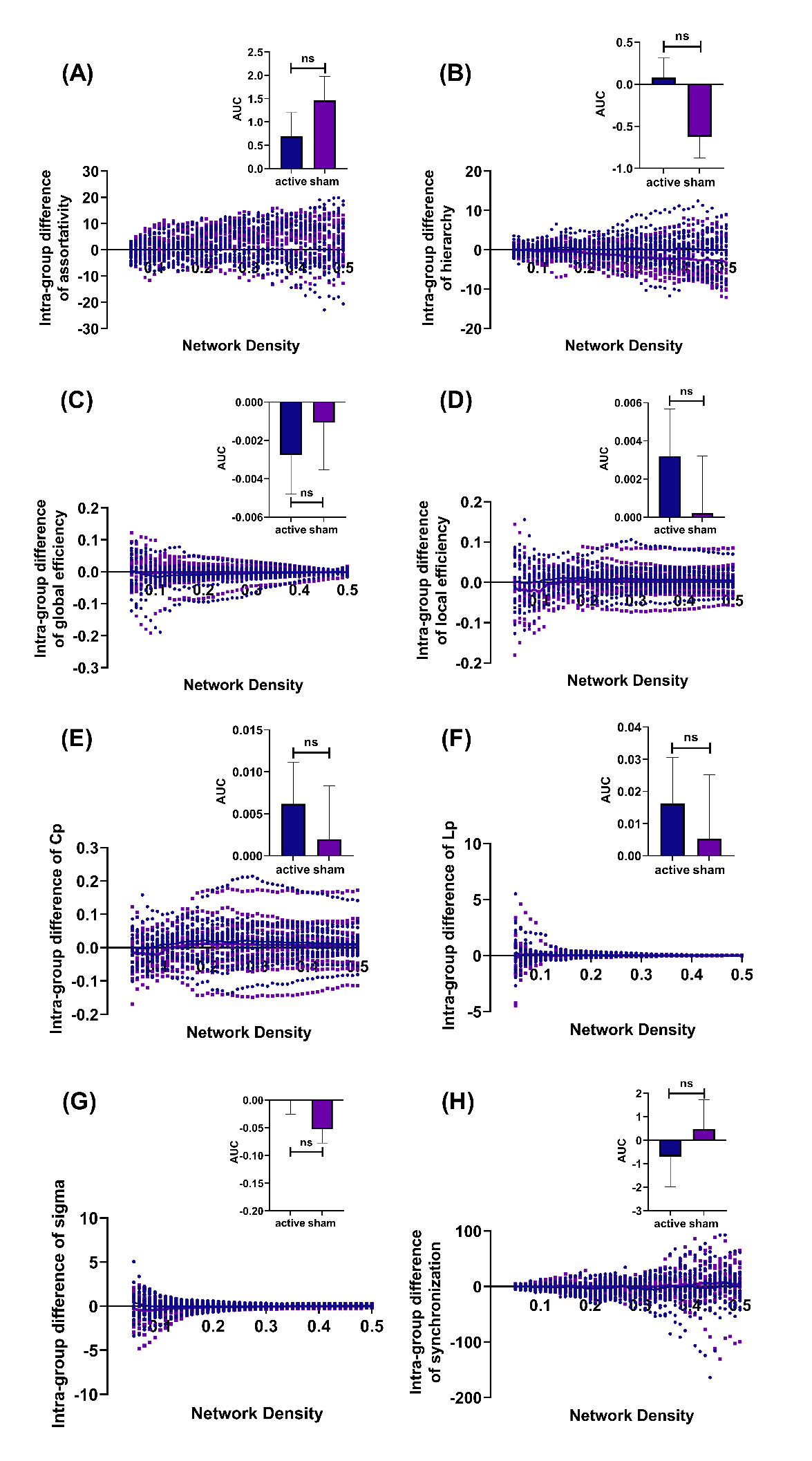


**Figure S2. The inter-group comparison of the global network properties.** There was no inter-group difference of AUCs of global network properties (all *P* > 0.05). The changes of assortativity (A), hierarchy (B), global efficiency (C), local efficiency (D), *C_p_* (E), *L_p_* (F), *sigma* (G), and synchronization (H) with increasing network density for all participants in two groups were shown. *E_glob_, global efficiency. E_loc_, local efficiency. C_p_, clustering coefficient. L_p_, characteristic path length. AUC, area under the curve. ns, no significance*.


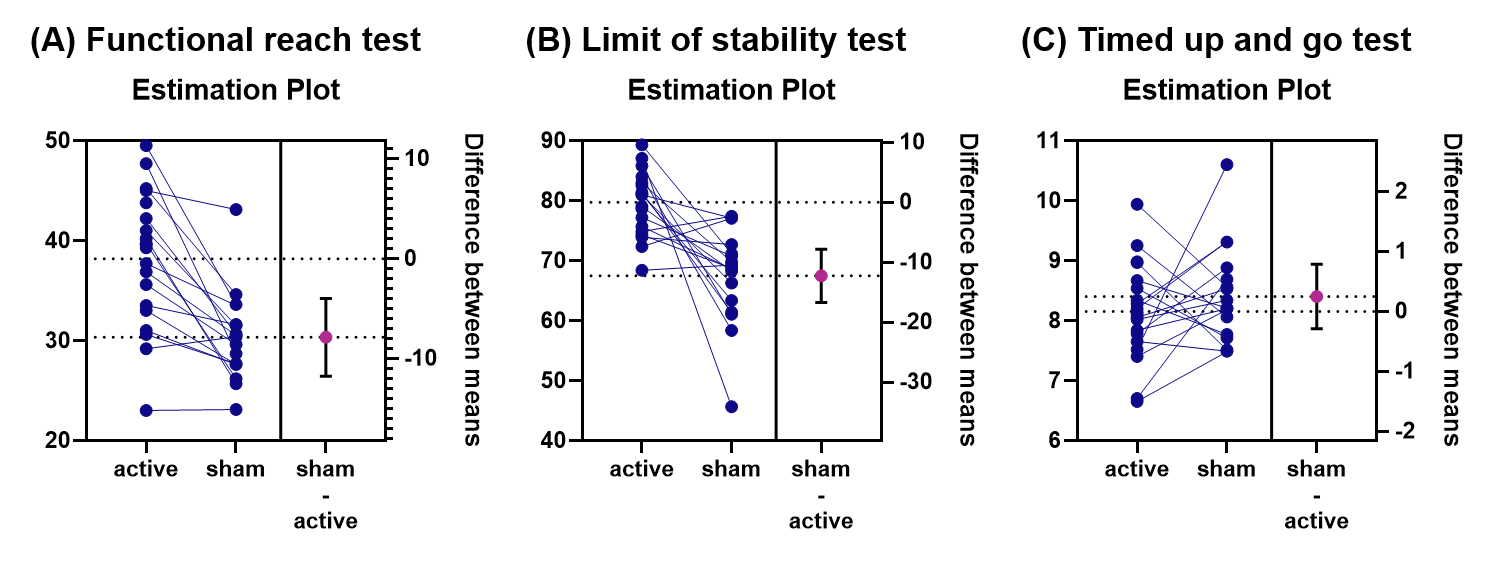


**Figure S3. The inter-group comparison of the balance tests.** The active ccPAS group had better performance in functional reach test (A) and limit of stability test (B) than the sham ccPAS group (all *P* < 0.05). There was no inter-group difference of timed up and go test (*P* > 0.05).


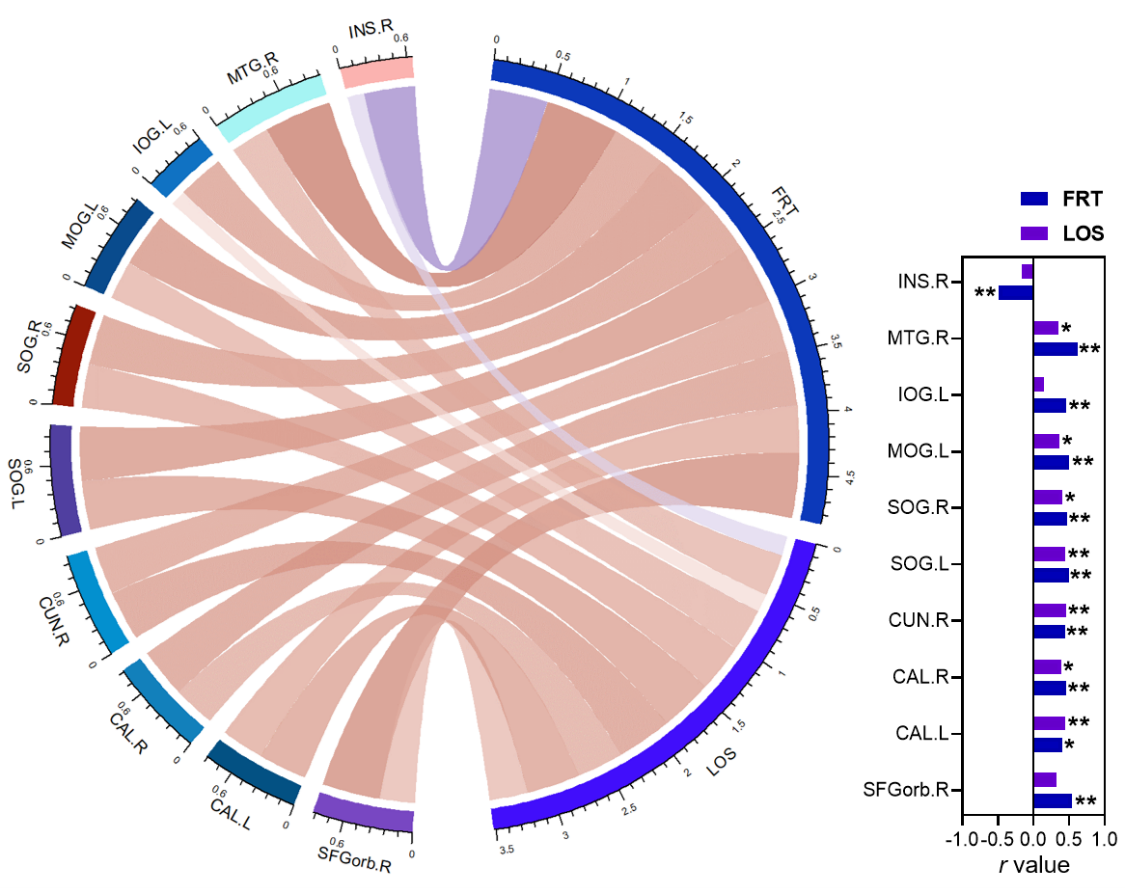


**Figure S4. The correlations between zfALFF and balance tests.** Red and blue lines in the left circle indicated positive and negative correlations, respectively. The thicker the line, the darker the color, and the greater the *r* values. *SFGorb, orbital part of superior frontal gyrus.* *CAL, calcarine. CUN, cuneus. SOG, superior occipital gyrus. MOG, middle occipital gyrus. IOG, inferior occipital gyrus. MTG, middle temporal gyrus. INS, insula. FRT, functional reach test. LOS, limit of stability. L, left, R, right*. *, *P* < 0.05; **, *P* < 0.01.

**Table S1. Safety screening questionnaire for rTMS**

| Safety screening questionnaire for rTMS |
| --- |
| 1. Do you have epilepsy or have you ever had a convulsion or a seizure? |
| 2. Have you ever had a fainting spell or syncope? If yes, please describe on which occasion(s)? |
| 3. Have you ever had a head trauma that was diagnosed as a concussion or was associated with loss of consciousness? |
| 4. Do you have any hearing problems or ringing in your ears? |
| 5. Do you have cochlear implants? |
| 6. Are you pregnant or is there any chance that you might be? |
| 7. Do you have metal in the brain, skull or elsewhere in your body (e.g., splinters, fragments, clips, etc.)? If so, specify the type of metal. |
| 8. Do you have an implanted neurostimulator (e.g., DBS, epidural/subdural, VNS)? |
| 9. Do you have a cardiac pacemaker or intracardiac lines? |
| 10. Do you have a medication infusion device? |
| 11. Are you taking any medications? (please list) |
| 12. Did you ever have a surgical procedure to your spinal cord? |
| 13. Do you have spinal or ventricular derivations? |
| 14. Did you ever undergo TMS in the past? If so, were there any problems. |
| 15. Did you ever undergo MRI in the past? If so, were there any problems. |

With reference to： *Rossi S, Hallett M, Rossini PM, Pascual-Leone A; Safety of TMS Consensus Group. Safety, ethical considerations, and application guidelines for the use of transcranial magnetic stimulation in clinical practice and research. Clin Neurophysiol. 2009, 120(12):2008-2039*.

**Table S2. The results of the inter-group comparison of the zfLAFF**

| Cluster No. | Brain regions | size  (voxels) | *T* value | MNI coordinates | | |
| --- | --- | --- | --- | --- | --- | --- |
|  |  |  |  | *x* | *y* | *z* |
| **active ccPAS group > sham ccPAS group** | | | | | | |
| 1 | left calcarine | 85 | 4.977 | -6 | -78 | 3 |
|  | right calcarine | 26 |  |  |  |  |
| 2 | right SOG | 53 | 4.392 | 24 | -90 | 30 |
|  | right cuneus | 7 |  |  |  |  |
| 3 | right MTG | 14 | 3.921 | -51 | -36 | 6 |
| 4 | left MOG | 77 | 3.786 | -30 | -99 | 3 |
|  | left SOG | 15 |  |  |  |  |
| 5 | left IOG | 10 | 3.551 | -51 | -75 | -9 |
| 6 | right SFGorb | 8 | 3.500 | 12 | 51 | -15 |
| **active ccPAS group < sham ccPAS group** | | | | | | |
| 7 | right insula | 16 | -3.625 | 42 | 15 | -3 |

*ccPAS, cortico-cortical paired-associative stimulation. SOG, superior occipital gyrus. MTG, middle temporal gyrus. MOG, middle occipital gyrus. IOG, inferior occipital gyrus. SFGorb, orbital part of superior frontal gyrus*.

**Table S3. The results of the inter-group comparison of the ROI-wise functional connectivity**

| Functional connectivity | active ccPAS group | sham ccPAS group | mean difference  (95% CI) | *T* | *P* |
| --- | --- | --- | --- | --- | --- |
| M1.R-STN.R | 0.120±0.262 | -0.039±0.166 | 0.159(0.010, 0.309) | 2.164 | 0.037 |
| SFG.L-IFGtri.L | 0.193±0.219 | -0.079±0.203 | 0.272(0.130, 0.414) | 3.898 | <0.001 |
| SFGorb.R-PCUN.L | 0.095±0.206 | -0.139±0.246 | 0.234(0.083,0.385) | 3.151 | 0.003 |
| SFGorb.R- PCUN.R | 0.069±0.213 | -0.149±0.203 | 0.218(0.079,0.358) | 3.180 | 0.003 |
| MFGorb.L-SFGmed.R | 0.092±0.195 | -0.127±0.192 | 0.219(0.090,0.349) | 3.435 | 0.002 |
| MFGorb.L- PCUN.L | 0.068±0.192 | -0.119±0.141 | 0.187(0.073,0.301) | 3.318 | 0.002 |
| IFGoperc.R- PCC.L | 0.218±0.172 | -0.057±0.311 | 0.274(0.110,0.439) | 3.387 | 0.002 |
| IFGorb.R- PCUN.L | 0.131±0.206 | -0.107±0.217 | 0.238(0.097.0.379) | 3.418 | 0.002 |
| PCC.L- IPG.L | 0.197±0.253 | -0.119±0.251 | 0.316(0.147,0.485) | 3.803 | 0.001 |
| PCC.L- IPG.R | 0.196±0.250 | -0.139±0.278 | 0.335(0.159,0.512) | 3.857 | <0.001 |
| CAU.L- GPi.L | -0.057 ± 0.243 | 0.104 ± 0.224 | -0.161(-0.319, -0.004) | -2.085 | 0.044 |
| PUT.L- PUT.R | -0.031 ± 0.259 | 0.194 ± 0.366 | -0.225(-0.434, -0.016) | -2.181 | 0.036 |

*ccPAS, cortico-cortical paired-associative stimulation. STN, subthalamic nucleus.* S*FG, superior frontal gyrus. IFGtri, triangular part of inferior frontal gyrus. SFGorb, orbital part of superior frontal gyrus. PCUN, precuneus. MFGorb, orbital part of middle frontal gyrus. SFGmed, medial part of superior gyrus. IFGoperc, opercular part of inferior gyrus. IFGorb, orbital part of inferior frontal gyrus. PCC, post cingulate cortex. IPG, inferior parietal gyrus. CAU, caudate. GPi, internal globus pallidus. PUT, putamen. L, left. R, right. CI, confident interval*.

**Table S4.** **The results of the inter-group comparison of global network properties**

| Properties | active ccPAS group | sham ccPAS group | mean difference  (95% CI) | *T* | *P* |
| --- | --- | --- | --- | --- | --- |
| Assortativity | 0.692±2.270 | 1.463±2.125 | -0.771 (-2.248, 0.705) | -1.060 | 0.296 |
| Hierarchy | 0.078±1.045 | -0.624±1.049 | 0.702 (0.001, 1.403) | 2.034 | 0.050 |
| *Eglob* | -0.003±0.009 | -0.001±0.010 | -0.002 (-0.008, 0.005) | -0.532 | 0.598 |
| *Eloc* | 0.003±0.011 | 0.000±0.012 | 0.003 (-0.005, 0.011) | 0.772 | 0.445 |
| *Cp* | 0.006±0.022 | 0.002±0.026 | 0.004 (-0.012, 0.020) | 0.531 | 0.599 |
| *Lp* | 0.016±0.064 | 0.005±0.082 | 0.011 (-0.038, 0.059) | 0.454 | 0.653 |
| *Sigma* | 0.000±0.113 | -0.053±0.102 | 0.053 (-0.020, 0.125) | 1.471 | 0.150 |
| Synchronization | -0.693±5.745 | 0.486±4.841 | -1.179 (-4.918, 2.560) | -0.642 | 0.525 |

Data are mean ± standard deviation. *ccPAS, cortico-cortical paired-associative stimulation. E_glob_, global efficiency. E_loc_, local efficiency. C_p_, clustering coefficient. L_p_, Characteristic path length. CI, confident interval.*

**Table S5. The results of the inter-group comparison of regional network properties**

| Network  properties | Brain regions | active ccPASgroup | sham ccPASgroup | mean difference  (95%CI) | *T* | *P* |
| --- | --- | --- | --- | --- | --- | --- |
| BC | IFGtri.L | 4.594±12.526 | -9.477±25.133 | 14.071(1.121, 27.021) | 2.206 | 0.034 |
|  | MCC.R | 15.800±20.387 | -2.273±26.936 | 18.073(2.263 ,33.883) | 2.321 | 0.026 |
|  | AMYG.R | -2.591±17.412 | 9.555±17.743 | -12.145(-23.908, -0.382) | -2.096 | 0.043 |
| NLp | IFGorb.R | 0.036±0.116 | -0.070±0.118 | 0.106(0.020, 0.193) | 2.519 | 0.018 |
|  | HIP.R | 0.143±0.328 | -0.171±0.348 | 0.314(0.065, 0.563) | 2.582 | 0.015 |

*ccPAS, cortico-cortical paired-associative stimulation. BC, betweenness centrality. NL_p_, nodal characteristic path length. IFGtri, triangular part of inferior frontal gyrus. MCC, middle cingulate cortex. AMYG, amygdala. IFGorb, orbital part of inferior frontal gyrus. HIP, hippocampus. L, left. R, right. CI, confident interval.***Table S6. The correlations between zfALFF and balance tests**

| zfALFF of  brain regions | FRT | | LOS | | TUG | |
| --- | --- | --- | --- | --- | --- | --- |
|  | *r* | *P* | *r* | *P* | *r* | *P* |
| right SFGorb | 0.541 | 0.001 | 0.323 | 0.051 | -0.013 | 0.937 |
| left calcarine | 0.400 | 0.014 | 0.450 | 0.005 | -0.173 | 0.305 |
| right calcarine | 0.460 | 0.004 | 0.391 | 0.017 | -0.207 | 0.218 |
| right cuneus | 0.443 | 0.006 | 0.464 | 0.004 | -0.275 | 0.099 |
| left SOG | 0.497 | 0.002 | 0.449 | 0.005 | -0.218 | 0.195 |
| right SOG | 0.468 | 0.004 | 0.406 | 0.013 | -0.186 | 0.271 |
| left MOG | 0.504 | 0.001 | 0.360 | 0.029 | -0.265 | 0.113 |
| left IOG | 0.453 | 0.005 | 0.153 | 0.365 | -0.414 | 0.011 |
| right MTG | 0.616 | < 0.001 | 0.356 | 0.030 | -0.27 | 0.106 |
| right insula | -0.490 | 0.002 | -0.164 | 0.333 | 0.286 | 0.086 |

*SFGorb, orbital part of superior frontal gyrus*. *SOG, superior occipital gyrus. MOG, middle occipital gyrus. IOG, inferior occipital gyrus. MTG, middle temporal gyrus.*

**Table S7. The correlations between ROI-wise FC and balance tests**

| Functional connectivity | | FRT | | LOS test | | TUG test | |
| --- | --- | --- | --- | --- | --- | --- | --- |
|  |  | *r* | *P* | *r* | *P* | *r* | *P* |
| 1 | M1.R-STN.R | 0.439 | 0.007 | 0.144 | 0.397 | -0.202 | 0.232 |
| 2 | SFG.L-IFGtri.L | 0.456 | 0.005 | 0.381 | 0.020 | -0.236 | 0.159 |
| 3 | SFGorb.R -PCUN.L | 0.399 | 0.015 | 0.294 | 0.077 | -0.104 | 0.541 |
| 4 | SFGorb.R - PCUN.R | 0.358 | 0.030 | 0.397 | 0.015 | 0.020 | 0.905 |
| 5 | MFGorb.L -SFGmed.R | 0.435 | 0.007 | 0.073 | 0.668 | -0.227 | 0.177 |
| 6 | MFGorb.L - PCUN.L | 0.468 | 0.004 | 0.128 | 0.449 | 0.035 | 0.837 |
| 7 | IFGoperc.R - PCC.L | 0.424 | 0.009 | 0.413 | 0.011 | -0.153 | 0.365 |
| 8 | IFGorb.R - PCUN.L | 0.481 | 0.003 | 0.252 | 0.132 | -0.223 | 0.184 |
| 9 | PCC.L - IPG.L | 0.435 | 0.007 | 0.288 | 0.084 | -0.078 | 0.647 |
| 10 | PCC.L - IPG.R | 0.422 | 0.009 | 0.284 | 0.088 | 0.039 | 0.821 |
| 11 | CAU.L – GPi.L | -0.464 | 0.004 | -0.185 | 0.273 | 0.071 | 0.678 |
| 12 | PUT.L – PUT.R | -0.321 | 0.053 | 0.018 | 0.915 | 0.133 | 0.433 |

*FRT, functional reach test. LOS, limit of stability. TUG, timed up and go. SFG, superior frontal gyrus. IFGtri, triangular part of inferior frontal gyrus. SFGorb, orbital part of superior frontal gyrus. PCUN, precuneus. MFGorb, orbital part of middle frontal gyrus. SFGmed, medial part of superior frontal gyrus. IFGoperc, opercular part of inferior frontal gyrus. IFGorb, orbital part of inferior frontal gyrus. PCC, posterior cingulate cortex. IPG, inferior parietal gyrus. CAU, caudate. GPi, internal globus pallidus. PUT, putamen. L, left. R, right.*
